# Application of Brown Planthopper Salivary Gland Extract to Rice Plants Induces Systemic Host mRNA Patterns Associated with Nutrient Remobilization

**DOI:** 10.1371/journal.pone.0141769

**Published:** 2015-12-07

**Authors:** Adelina Petrova, Charles Michael Smith

**Affiliations:** 1 School of Biological Sciences, Washington State University, Pullman, WA, United States of America; 2 Department of Entomology, Kansas State University, Manhattan, KS, United States of America; University College Dublin, IRELAND

## Abstract

Insect saliva plays an important role in modulation of plant-insect interactions. Although this area of research has generated much attention in recent years, mechanisms of how saliva affects plant responses remain poorly understood. To address this void, the present study investigated the impact of the brown planthopper (*Nilaparvata lugens*, *Stål;* hereafter BPH) salivary gland extract (SGE) on rice (*Oryza sativa*) systemic responses at the mRNA level. Differentially expressed rice mRNAs were generated through suppression subtractive hybridization (SSH) and classified into six functional groups. Those with the most representatives were from the primary metabolism (28%), signaling-defense (22%) and transcription-translation-regulation group (16%). To validate SSH library results, six genes were further analyzed by One-Step Real-Time Reverse Transcriptase-PCR. Five of these genes exhibited up-regulation levels of more than 150% of those in the control group in at least one post-application time point. Results of this study allow assignment of at least two putative roles of BPH saliva: First, application of SGE induces immediate systemic responses at the mRNA level, suggesting that altering of the rice transcriptome at sites distant to hoppers feeding locations may play an important role in BPH-rice interactions. Second, 58% of SGE-responsive up-regulated genes have a secondary function associated with senescence, a process characterized by remobilization of nutrients. This suggests that BPH salivary secretions may reprogram the rice transcriptome for nutritional enhancement. When these findings are translated onto ‘whole plant’ scale, they indicate that BPH saliva may play the ‘wise investment’ role of ‘minimum input today, maximum output tomorrow’.

## Introduction

Plants have a complex response network that enables them to cope with both biotic and abiotic factors. Plant responses to phloem-feeding insects (aphids, whiteflies, hoppers) are the result of all insect activities. However, feeding has the greatest impact on plant metabolism, morphology and gene expression [1–8]. Phloem-feeding is a complex process that consists of four distinct phases—exploration, probing, salivation and ingestion of plant nutrients. Each of these phases serves a specific role that has a unique impact on plant responses. While exploration and probing might cause mechanical damage, salivation and ingestion of phloem sap have a more complex impact on the host plant [1, 2].

Phloem-feeding insects have evolved two types of saliva (stylet sheath and watery saliva) to facilitate feeding strategies [9]. The stylet sheath is a proteinaceous viscous mixture that upon secretion polymerizes rapidly to form a tubular structure. This straw-like formation provides mechanical aid to the stylet and retains the integrity of the plant cell membrane during probing and feeding [9, 10]. Several enzymes such as polyphenol oxidases and peroxidases are infused in the salivary sheath and may participate in the suppression of plant defense responses [11, 12]. Watery saliva, secreted during probing and ingestion of phloem sap, contains different types of oxidoreductases (peroxidases, polyphenol oxidases) and hydrolases (pectinases, cellulases) that may play important roles in overcoming defense responses as well as detoxifying secondary metabolites in the host plant [9, 13, 14].

To date, only a few studies have elucidated the role of phloem-feeding insect saliva on plant responses. One of these studies suggested that the presence of calcium-binding proteins from aphid saliva might modulate calcium-sensitive sieve-tube occlusion mechanisms [15]. This statement is supported by *in-vitro* experiments data where extracted forisomes responded to application of aphid salivary compounds. Forisomes are proteinaceous inclusions in the sieve tubes of legume plants, whose ‘open’ or ‘closed’ state is known to respond to calcium concentrations [16, 17]. Since saliva-treated forisomes do assume a non-plugging state [15], it is presumed that in *in-vivo* settings this mechanism will ensure a continuous flow of phloem sap. However, it is likely that *in-vivo* interactions between aphid saliva and phloem proteins are more complex. For instance, Ca^2+^ oscillations are closely connected with Reactive Oxygen Species (hereafter ROS), [18]. Therefore, secreted salivary oxidoreductases will impact plant ROS levels [2, 9, 19], which in turn may affect Ca^2+^ oscillations and thus, the status of forisomes.

Investigations elucidating the impact of phloem-feeding insect saliva on host responses are limited. A study of *Myzus persicae* (green peach aphid) demonstrated that application of salivary compounds to *Arabidopsis thaliana* foliage induces local host defense responses which are independent of currently known signaling pathways (salicylic acid, jasmonate and ethylene) [20]. It is important to highlight that this study recorded *Arabidopsis thaliana* responses to salivary secretions that have been collected from artificial diets after aphid feeding. This approach has two potential limitations. First, since aphid salivary spectrum varies according to its nutrient type (e.g. susceptible/resistant host plants, artificial diets) [9], it is likely that the salivary secretions collected from artificial diets will differ from those when aphids feed on plants. Second, since collection of salivary compounds is carried out over an extended period of time, degradation processes may affect sample quality. The alternative approach, which was adopted by this study, is the direct extraction of salivary glands. It allows obtaining a wider spectrum of salivary compounds and, most importantly, degradation processes are significantly reduced, since dissection procedures are rapid and carried out on ice.

Brown planthopper (*Nilaparvata lugens*, hereafter BPH) is an important pest of rice that feeds on sap from the vascular bundles (phloem and xylem), but predominantly phloem, and causes damage by removing nutrients and transmitting viruses. During the initial stages of BPHs feeding, no apparent changes in the host plant are observed. However, heavy infestations cause senescence-like symptoms (yellowing), followed by complete drying of the plant, also known as ‘hopperburn’ [21, 22]. BPH transmits economically important viruses such as rice grassy stunt virus (RGSV) and rice ragged stunt virus (RRSV) [23]. Use of BPH-resistant rice varieties is the most effective method of controlling BPH populations [24]. Although several BPH-resistant rice genes have been introduced into rice varietal production [25], the exact molecular mechanisms of resistance are not known. Thus, dissecting rice responses to BPH, including defence and signaling networks, is an important step towards understanding the molecular basis of resistance and the development of new strategies for control of BPH populations.

**Fig 1:**
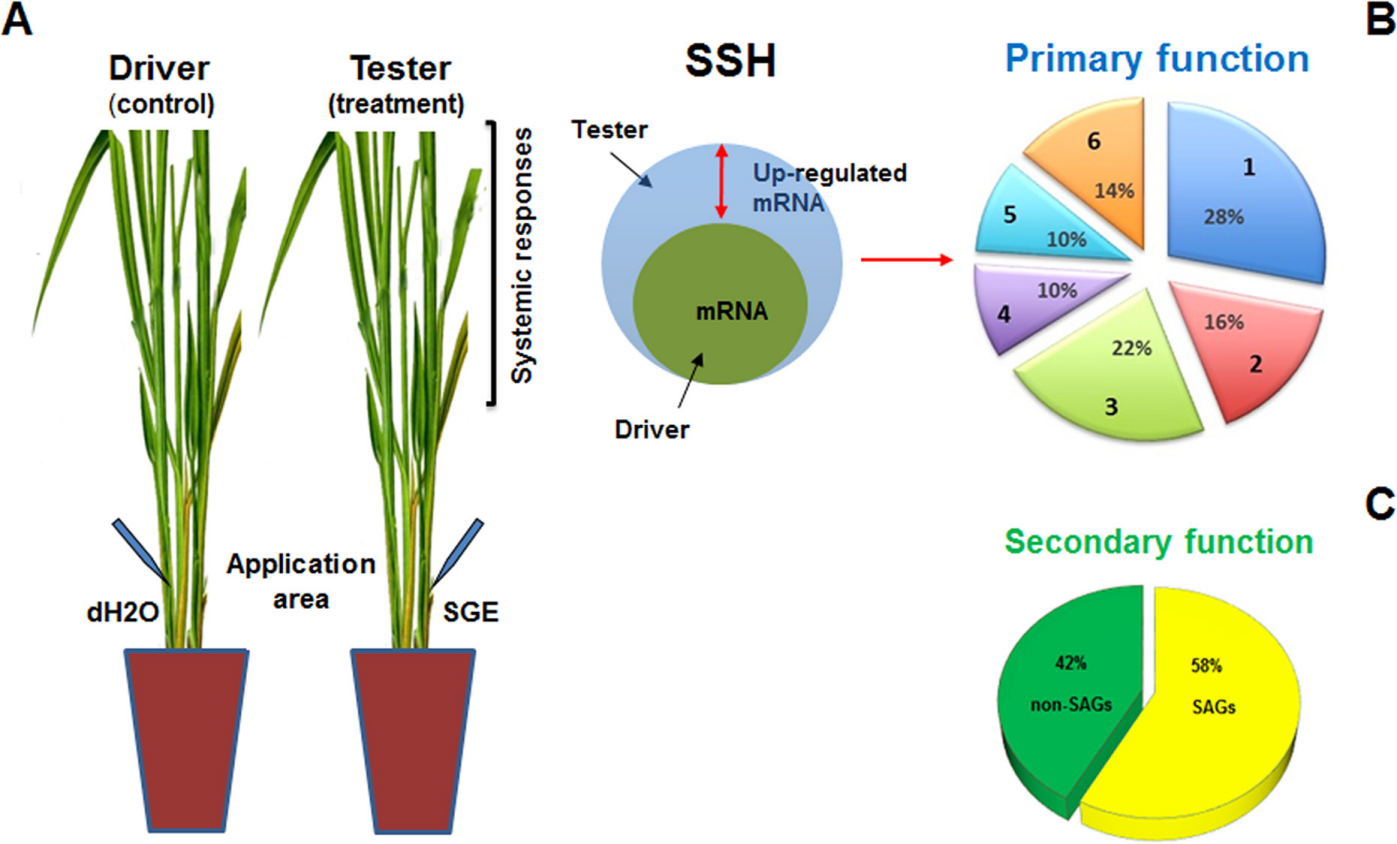
Experimental plot (A) and functional classification of BPH SGE-responsive systemically up-regulated genes in TN1 rice variety (B-C). Visual representation of the experimental plot and the principle of suppression subtractive hybridization (SSH) is shown in (A). According to their primary function ESTs from the up-regulated gene pool were classified into six functional groups (B): 1-primary metabolism, 2-transcription/ translation/regulation, 3-signaling/defense, 4-transport, 5-other, and 6-unknown. Further classification (C) was carried out to identify ESTs related to previously studied orthologous senescence associated genes (SAGs).

As described above, host plant responses are the result of all insect activities (probing, salivating, ingesting sap and nutrients, etc.). Each of these has a specific effect and impact on the host plant. Therefore, understanding the role of each enables pinpointing of key molecular mechanisms in plant resistance. The primary focus of this study was to understand how BPH saliva affects rice systemic responses at the mRNA level. Since there is no precise way to study insect saliva and its effects on the host plant, the current study has adopted the ‘closest’ methodology by applying BPHs salivary gland extract (hereafter SGE) to the rice plant. SGE application was accompanied by fine-needle piercing of the host tissue to mimic BPHs stylet probing and saliva delivery into the host plant. In contrast, in the control group of rice plants, distilled water was applied instead of SGE. Subtractive hybridization was carried out to identify SGE-responsive systemically up-regulated genes from the tissue of Taichung Native 1 (TN1), a BPH-susceptible rice variety (Fig 1A). The results of transcript analyses point out that SGE application to rice plants modulates host mRNA levels systemically in such a way that may potentially enhance the host nutritional status (Fig 1B and 1C) [26–31].

## Materials and Methods

### Plant material

Seeds of the rice variety Taichung Native 1 (susceptible to BPH) were obtained from the International Rice Research Institute (IRRI), Philippines, courtesy of G. S. Kush. Each plant was grown individually in a single pot (under non-flooding conditions), in a controlled environment room, at a temperature range 20–30°C (night/day) and L12:D12 (light/dark) photoperiod [1, 2]. Four to five week old plants were used for all experiments.

### Insects

BPHs held under Defra Licence PHL 163/5509 (11/2006) were reared on ‘Taichung Native 1’ 8-week-old rice plants in controlled environment conditions at a temperature range of 22–28°C (night/day) and L16:D8 (light/dark) photoperiod. BPHs have been reared under these conditions for several years and are regarded as a laboratory strain.

### Salivary extract

Female BPH adults were anaesthetised by cooling on ice and salivary glands were dissected in cold 1 x PBS buffer. Salivary gland tissue was homogenized with a glass rod in distilled water. Each batch of tissue contained 30–35 salivary glands, and was homogenized with 90 μl of distilled water. The homogenates were centrifuged at 14,000 *g* for 15 min at 4°C. The supernatants were collected, frozen in liquid N_2_ and placed in short-term storage at -20°C.

### BPH saliva bioassay

Control plants in this bioassay were mechanically injured by piercing the ‘stem’ (basal part) 10 cm above the plant base with 1 mm capillary needle (Fig 1A). Plants were pierced 100 times in small circular patterns (20 circles X 5 needle piercings) and 4.5 μl of distilled water was applied to each circle (90 μl in total). In the experimental group, mechanical injury was combined with an application of SGE-soluble fraction. For each plant, 90 μl of extract was applied, which was the equivalent of 30–35 salivary glands. To study systemic responses, leaf tissue (all leaves) from the control and experimental groups was collected separately at 0.5-, 4-, 24-, 48- and 72 h post-application. Samples were frozen in liquid N_2_, powdered with a pre-cooled pestle and mortar and stored at -80°C. Seven plants were used for each biological replication for each group (control/experimental) and for each post-application time point. The entire experiment was repeated three times (7 plants/n; 3n = 21 plants for each time point of each group).

### RNA extraction, DNase treatment and RNA quantification

Leaf tissue from each time point and treatment was homogenized into liquid N_2_. Total RNA was extracted using TRI-Reagent (Helena Bioscience) by following the manufacturer’s instructions. RNA was resuspended in 0.15% DEPC treated water and stored at -80°C. In order to remove any genomic DNA contamination, samples were treated with a DNase I enzyme kit (Invitrogen) following the manufacturer’s instructions. For RNA quantity and quality analyses, absorbance values at 260 nm and 280 nm (A_260_, A_280_) were determined. The concentration of RNA was calculated from A_260_ values (A_260_ = 1 corresponds to a concentration of 40 μg/ml). The ratio A_260_/A_280_ was used to assess total RNA purity. For the SSH library, 150 μg of total RNA was taken from each time point (50 μg/n x 3n = 150 μg) and mixed together (5 time points x 150 μg total RNA = 750 μg total RNA) so that each group (control and experimental) contained 750 μg of total RNA (tissue from 105 plants; 3 biological replications). mRNA was purified using a PolyATtract mRNA kit (Promega). mRNA precipitation was carried out overnight at -20°C in 0.1 volume of 3 M sodium acetate (pH 5.2) and 1 volume of isopropanol. For One-Step Real-Time Reverse Transcriptase-PCR (hereafter, qPCR) total RNA was extracted from rice tissue of each biological replication (n = 3; 7 plants/n) and each post-application time point (0.5-, 24-, 72 h).

### Suppression subtractive hybridization (SSH)

SSH was carried out using the PCR-Select Subtractive Hybridization Kit (Clontech), [32]. Experimental and control samples for each treatment were processed simultaneously to reduce false positives. The amount of mRNA was increased to 4 μg instead of 2 μg as recommended by the manufacturer. To isolate fragments corresponding to up-regulated rice genes following SGE treatment, cDNA prepared from the experimental group (SGE + mechanical injury) was used as the ‘tester’, and cDNA from the control group (mechanical injury + water) as the ‘driver’ (Fig 1A). The reverse subtraction was performed to isolate fragments corresponding to genes whose expression levels were down-regulated following SGE treatment. For each subtraction, two rounds of PCR amplification were performed according to the manufacturer's protocol to enrich differentially-regulated genes.

### Cloning, transformation and plasmid purification

The secondary PCR products of the SSH were cloned into a pCR^®^2.1-TOPO vector and transformed into TOP10 *E*.*coli* competent cells (Invitrogen) following the manufacturer's instructions. About 200 colonies were obtained from each subtraction experiment and 100 randomly picked single colonies were grown overnight in 5 ml of liquid LB medium with the appropriate antibiotic. Plasmid DNA purification was carried out using a QIAfilter Plasmid Midi Kit (Qiagen).

### Restriction enzyme analyses, sequencing, sequence and functional analyses

Purified plasmids were subjected to restriction digestion with *Eco*RI (Fermentas). Digested plasmids were analysed on 1.6% agarose gels and visualised under UV illumination using a UVITEC Gel Doc System. Inserts ranged from ~150 to ~1500 bp in length, but the majority were ~500 bp. Sequencing was carried out using a T7 promoter primer. Each sequence was edited to correct sequencing ambiguities and to remove the primer sequence, followed by analyses at http://rice.plantbiology.msu.edu, using Blastx. Search for orthologous genes associated with senescence was carried out either through literature search or through Leaf senescence database (http://www.eplantsenescence.org/blast.php).

### Primer synthesis and sequences

Specific primers were manually designed and synthesized by Sigma-Genosys. Information on primer sequences and accession numbers of gene nucleotide sequences is shown in S1 Table.

### Validation of forward SSH library by qPCR

To validate the forward SSH library, six expressed sequence tags (hereafter, ESTs) were chosen for further expression analyses by qPCR. Transcript profiles of glutamine synthetase (*GS*), asparagine synthetase (*AS*), glyoxalase I (*Glyo I*), amino acid transporter (*AAT*), calcium/proton antiporter (*CaPA*) and dehydroascorbate reductase (*DHAR*) genes were examined in the control and the experimental tissue at 0.5-, 24- and 72 h post-application. For each selected time point, three biological replications were investigated (n = 7 plants). Two qPCRs for each biological replication were carried out, resulting in a total of 6 readings for each time point. qPCRs were carried out using a LightCycler RNA Master SYBR Green I kit (Roche Applied Science). In essence, the reaction was carried out in a 20 μl total volume, containing 1 X LightCycler RNA Master SYBR Green I master mix, 5 mM manganese acetate (Mn(OAc)_2_), 0.3 μM specific primers and total RNA template. Information on the amount of total RNA template used for each gene is included in S1 Table. The expression of the *18S* was investigated to assess RNA quality and quantity used in these studies. qPCRs with different cytokine RNA dilutions (LightCycler^®^ Control Kit RNA, Roche Applied Science) were carried out in parallel to provide both absolute gene quantification values and qPCR replication efficiency values. All reactions were carried out on a Roche LightCycler under the following conditions: one cycle at 52°C for 30 min, one cycle at 95°C for 40 s, followed by 40 cycles at 95°C for 10 s, 59°C for 12 s, 72°C for 20 s; concluded by one cycle at 72°C for 20 s and one cycle at 40°C for 30 s.

### Analyses of relative gene expression

Cycle threshold (Ct) values were exported to Excel and relative gene expression was calculated according to the 2^-ΔΔCt^ method [33]. Cycle threshold (Ct) values for the *18S* were used to normalize the expression patterns of the analyzed genes from control and treatment plants. The differences between the control and experimental group are presented as relative values. Based on previous literature, relative values above 1.5-fold (150% difference from the control) were regarded as biologically significant [20, 34].

### Statistical analyses

All results were tested for statistical significance using an unpaired Student’s t-test (*P*<0.05; Excel, Microsoft Office 2010).

## Results

### SSH Sequence analyses

**Fig 2:**
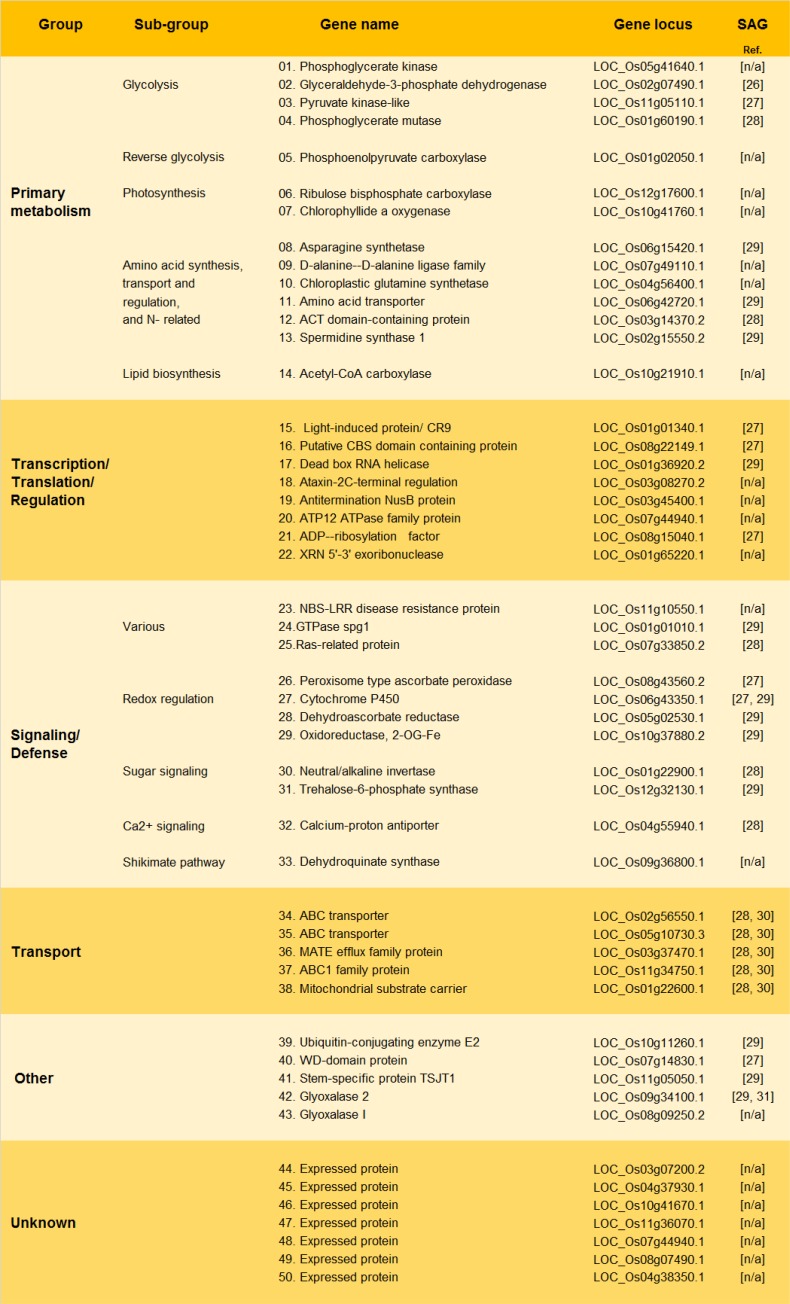
BPH SGE-responsive systemically up-regulated genes in TN1 rice variety. Reference information for genes related to previously studied SAGs is included.

Following restriction digestion, a total of 160 clones (80 from each forward and reverse subtraction) were randomly selected for sequencing. After removing redundant sequences, 50 and 60 ESTs were obtained for the forward (SGE-responsive systemically up-regulated genes) and reverse subtractions (SGE-responsive systemically down-regulated genes), respectively. ESTs from the two groups did not overlap, indicating the reliability of the two libraries. ESTs present in the forward SSH library were classified into six major functional groups, using information from various sources (Figs 1 and 2). The largest group contained 14 ESTs (28%) whose functions are related to primary metabolism. Within this group, ESTs were organized into several sub-groups, of which that of the nitrogen metabolism and the glycolysis had the most representatives. The group of ‘transcription-translation-regulation’ functions contained 8 ESTs (16%). ESTs from the following group (11 ESTs; 22%) are associated with signaling-defense roles, and those with redox functions had the most representatives. Two groups–those of ‘transport’ and ‘other’ functions had 5 ESTs each (10%), and 7 ESTs with unknown functions (14%) were classified into a separate group. From the total of 50 SGE-responsive systemically up-regulated ESTs, 29 (58%) are related to orthologous senescence associated genes (hereafter SAGs), (Fig 2).

Although the primary goal of this study was to investigate SGE-responsive systemically up-regulated rice genes, functional classification of the down-regulated mRNA pool is presented in S2 Table which also includes reference information for SAGs [28, 29, 35]. Comparative analyses between ESTs functional groups of the up-regulated and the down-regulated pools is shown in S1 Fig.

### Analyses of selected forward SSH library ESTs by qPCR

**Fig 3:**
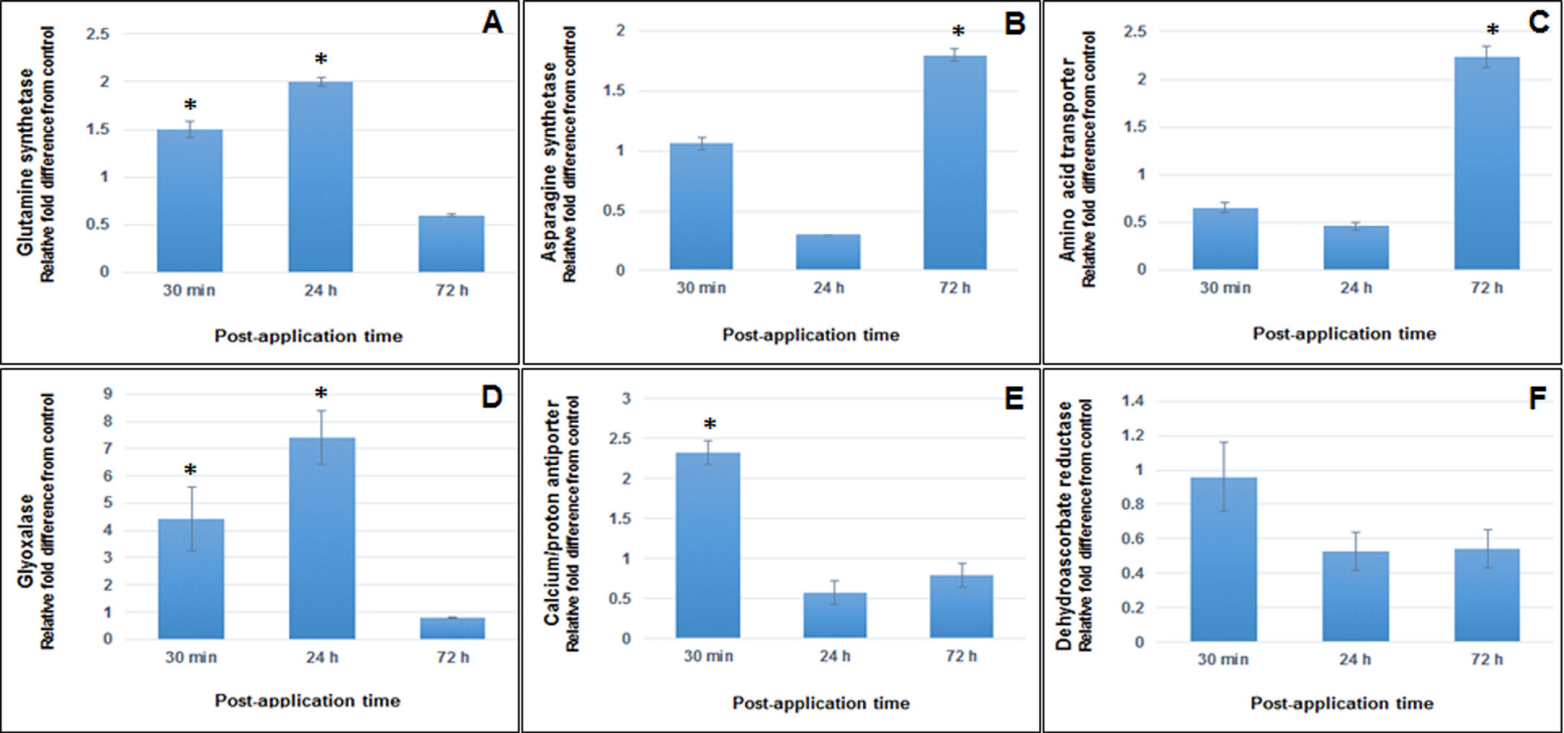
Transcript profiling of six BPH SGE-responsive systemically up-regulated genes in TN1 rice variety. Expression profiles of glutamine synthetase (A), asparagine synthetase (B), amino acid transporter (C), glyoxalase I (D), calcium/proton antiporter (E) and dehydroascorbate reductase (F) genes were investigated in the control and experimental tissue of TN1 rice variety at 0.5-, 24- and 72 h post-application. Values are the mean of three biological replications; asterisks indicate significant difference between gene expression levels of SGE-treated and non-treated rice plants (n = 3; SE±; *P<0.05, Student's t-test).

Gene expression patterns of glutamine synthetase (*GS*), asparagine synthetase (*AS*), glyoxalase I (*Glyo I*), amino acid transporter (*AAT*), calcium/proton antiporter (*CaPA*) and dehydroascorbate reductase (*DHAR*) genes were examined in the control and the experimental tissue at 0.5-, 24- and 72 h post-application. Transcriptome analyses of each gene demonstrated unique ‘signature’ response (Fig 3). *GS* responded to SGE application at 0.5- and 24 h post- application with 1.5- and 2-fold up-regulation (compared to the control) (*P* = 0.003, *P* = 0.002, Student’s t-test), (Fig 3A). In contrast, *AS* expression levels were affected at a later stage (72 h post-application) by a 1.8-fold increase (compared to the control) (*P* = 0.0003, Student’s t-test), (Fig 3B). Similar to *AS* response, *AAT* had a 2.3-fold increase at 72 h post-application (compared to the control; *P* = 0.007, Student’s t-test), (Fig 3C). BPHs SGE had the most impact on the expression patterns of *Glyo I* with 4.4- and 7.4-fold increases (compared to the control) at 0.5- and 24 h post-application, respectively (*P* = 0.04, *P* = 0.01, Student’s t-test), (Fig 3D). Ca^2+^ fluxes are associated with initiation of host cell responses, also observed in *CaPA* expression levels by 2.3-fold increase (compared to the control) at 0.5 h post-application (*P* = 0.008, Student’s t-test), (Fig 3E). *DHAR* was the only gene that had no significant response following SGE treatment (*P*>0.05, Student’s t-test), (Fig 3F).

## Discussion

This study investigated BPHs SGE-mediated effects on the rice transcriptome so as to understand the role of hoppers saliva in altering rice responses (Fig 1). Among several important conclusions, two have emerged to form a milestone in this study. First, BPHs SGE modulates rice transcriptome at sites distant to hoppers feeding locations, suggesting a systemic effect of saliva. Usually, hoppers feed along the basal part of the rice plant. Therefore, BPHs SGE was applied in the basal part of each rice plant and the leave tissue was analysed to understand the systemic effects (Fig 1A). The fact that BPHs SGE induces immediate systemic effects underlines the significance of this mechanism in BPH-rice interactions. Second, application of BPHs SGE to rice plants alters the expression of numerous mRNAs associated with nutrient turnover (Fig 1B and 1C). In the up-regulated pool, 28% of ESTs have putative functions associated with plant primary metabolism, including amino acids synthesis and transport (Fig 2). Furthermore, 58% of the up-regulated and 28% of the down-regulated ESTs have similar expression patterns to orthologous SAGs during senescence (S1 Fig). Taken together, these results suggest the putative role of BPHs saliva in modulation of rice nutrient physiology by altering transcription levels of genes involved in senescence-like mechanisms.

Senescence is a highly regulated process, characterized by controlled catabolism of macromolecules and remobilization of materials [26]. Some phloem-feeding insects induce senescence-like symptoms in the host plant during infestation. Such symptoms might correlate with nutritional enhancement by altering amino acid composition and levels in the host tissue following aphids [36–38], or BPHs infestation [1]. However, to date, there is no direct evidence elucidating the mechanism of this phenomenon. Remobilization of nutrients will enhance the nutritional status of the phloem sap, which is the main nutrient source for phloem-feeding insects. Nitrogen is a growth-limiting factor for both plants and insects, and its availability will profoundly influence the development and survival of both sides. Rice phloem sap, which is the primary feeding source for the BPH, has low nitrogen (free amino acids 3–8% w/v, mainly glutamine, glutamate, asparagine and aspartate) and high sucrose concentrations (17–25% w/v) along with ATP, minerals and organic acids [39–41].

Application of BPHs SGE to the rice plant stem up-regulated the systemic expression of glutamine synthetase (*GS*) and asparagine synthetase (*AS*) genes (Fig 3A and 3B), which code for enzymes catalysing the biosynthesis of glutamine and asparagine, respectively. During senescence, both glutamine and asparagine are the predominant types of amino acids in the phloem sap. Their role in nitrogen remobilization is well-demonstrated [42, 43]. In this study, the up-regulated *GS* codes for the chloroplastic iso-form and, to date, there are no reports connecting this iso-gene to senescence. However, it is likely that different factors inducing senescence will initiate specific mechanisms. Further, processes occurring in the initial stages of senescence may differ from those taking place in the later stages [29]. Thus, the mechanism by which saliva alters nutrient remobilization in its host plant is likely to be unique. The primary role of the chloroplastic GS (EC 6.3.1.2) iso-form is re-assimilation of photorespiratory ammonium (NH_4_^+^). In C_3_ plants such as rice, photorespiratory ammonium release may exceed the primary nitrogen assimilation by a factor of 10 [44]. Barley (*Hordeum vulgare*) mutants defective in chloroplastic *GS2* are unable to re-assimilate photorespiratory ammonium and die when grown under normal conditions [45]. Therefore, increased activity of the chloroplastic GS iso-form is likely to enhance re-assimilation of photorespiratory ammonium, leading to higher nitrogen use efficiency. From the hopper’s perspective, this would mean an improved nutrient source.

Nitrogen remobilization during senescence requires efficient transport mechanisms facilitated by amino acid transporters. These have essential roles in nitrogen relocation and partitioning [46, 47] and belong to multiple gene families [48]. The fact that several transporters, including an amino acid transporter (*AAT*), are part of up-regulated ESTs pool provide additional evidence for nutritional enhancement in the host plant. qPCR expression analyses confirmed 2.3-fold *AAT* up-regulation (compared to the control) at 72 h post-application (Fig 3C), which also coincided with *AS* similar response patterns (1.8-fold increase at 72h post-application). Future studies will investigate correlations between the expression modes of these genes and determine their role and significance in BPH-rice interactions.

Further evidence for nutrient enrichment is the presence of the peptidyl-prolyl cis-trans isomerase gene in the down-regulated ESTs pool (S2 Table). The role of this enzyme is to regulate intracellular pH (pHi) homeostasis and protein folding. Suppression of gene(s) coding for this enzyme results in intracellular acidification [49]. Studies have shown that decreased pHi may affect nutrient cell status by altering the expression of nutrient–H^+^ symporters, including ABC transporters [49]. Interestingly, several genes coding for ABC transporters are found in the up-regulated ESTs pool. Current findings are consistent with previous results demonstrating an increase of free amino acids in the BPH-susceptible, but not in the BPH-resistant varieties after hoppers infestation [1]. Furthermore, senescence-like symptoms are more readily observed in infested BPH-susceptible varieties [1]. In contrast to the current understanding of the correlation between senescence and plant susceptibility to insects, a recent study suggested that senescence-like symptoms in *Arabidopsis thaliana* are associated with basal resistance towards the green peach aphid [50]. Therefore, it must be pointed out that although phloem-feeding insects have a common feeding site, feeding mechanisms among these species may differ. In addition, each plant species/variety has a specific and unique response network.

Four genes coding for enzymes involved in glycolysis are present in the up-regulated ESTs pool. Glycolysis is the first phase of respiration which has several functions in plant metabolism. Apart from being an important step in carbon flow (each hexose molecule is converted to two molecules of pyruvate), glycolysis generates energy (ATP) as well as reducing power (NADH) and intermediates that are utilized for biosynthesis of various products, including amino acids [51]. Degradation of glucose may be carried out through an alternative pathway from that of glycolysis, where no energy is produced and formation of methylglyoxal occurs [52]. Methylglyoxal is a product of protein and lipid catabolism as well. The ability of methylglyoxal to interact with biological macromolecules (DNA, RNA, proteins) and produce free radicals defines it as a highly reactive and toxic molecule [52]. Detoxification of methylglyoxal in the cell is carried out by the glyoxalase system, consisting of glyoxalase I and glyoxalase II enzymes (Glyo I and Glyo II) [53]. ESTs for both *Glyo I* and *Glyo II* were found in the systemically up-regulated mRNA pool, and further transcriptome analyses of *Glyo I* demonstrated 4- and 7-fold increase in SGE-treated tissues (compared to the control) at 0.5- and 24 h, respectively (Fig 3D). Up-regulation of genes from the glyoxalase system may have different effects that lead to similar physiological benefits. First, methylglyoxal is likely to be toxic to BPHs. Therefore, up-regulation of these genes suggests that SGE might stimulate the detoxification of methylglyoxal in the host cell. Second, an increase of methylglyoxal levels is an indication of protein, lipid and carbohydrate catabolism, which is a main feature of senescence-like processes. Differential expression of *Glyo II* is associated with senescence in *Arabidopsis thaliana*, whose possible physiological role is to maintain cell viability by conversion of methylglyoxal to D-lactic acid [31]. Third, transgenic tobacco overexpressing the glyoxalase pathway enzymes (*Glyo I* and *Glyo II*) exhibits enhanced tolerance to salinity and heavy metals [53–56], indicating the significance and role of these enzymes during stress. Thus, it is reasonable to speculate that BPHs saliva may have a putative role involved in sustaining rice cell viability during hoppers feeding.

Several mRNAs found in the up-regulated ESTs pool code for proteins involved in signaling and defense. Among these is the *CaPA* (calcium/proton antiporter), whose expression analyses confirmed over 2-fold increase (compared to the control) at 0.5 h post-application (Fig 3E). The activation of this gene is an indication of calcium (Ca^2+^) involvement in response to BPH SGE. In plants, Ca^2+^ is an important second messenger [57, 58] involved in response to both biotic (pathogens) and abiotic factors (temperature, light, drought, touch) [59–63]. Ca^2+^ oscillations are closely coordinated with ROS and cell redox homeostasis [18]. The presence of several ESTs coding for oxidoreductases (both libraries) suggests that BPH-rice redox interactions are an important factor in this relation. This statement is corroborated by previous studies on BPHs saliva, where catalase-like protein was detected in the infested BPH-resistant, and not in the susceptible variety [19]. Further, the same study demonstrated over 300% increase of catalase activity in the salivary glands of hoppers fed on BPH-resistant rice variety than those fed on BPH-susceptible variety. Thus, apart from highlighting the importance of the redox interactions between BPH and rice, these results have provided strong evidence of how nutrient source may affect insect salivary spectrum.

*DHAR* was the only gene from the analyzed group in which expression levels were below 1.5-fold (Fig 3F). Although expression profiles did not meet the significance thresholds (both statistical P<0.05 and biological 1.5-fold), it is important to analyze and interpret results in relation to their physiological aspect. DHAR (EC 1.8.5.1) is a part of the ascorbate-glutathione cycle (AA-GSH), whose mode of action is to reduce dehydroascorbate (DHA) to ascorbate (AA) with glutathione (GSH) as an electron donor [64]. At sub-cellular level, the AA-GSH cycle is the most important hydrogen peroxide detoxifying system in chloroplasts, peroxisomes and mitochondria that functions within finely-tuned ranges [65, 66]. Thus, small responses in *DHAR* expression levels may have significant physiological impacts.

Elucidating the effects of saliva from phloem-feeding insects on host plant responses is a challenging task, since mimicking the mechanism of saliva delivery (quantity, spectrum, spatial and temporal effects) to the host is practically impossible. Three important rationales constituted the core of the experimental design: First, the mechanical injury caused by insertion of BPH stylet during probing and feeding was simulated by fine needle piercing. Second, salivary compounds secreted during probing and feeding affect the mechanical injury impact on plant responses. This interaction was interpreted into the experimental design by introducing both mechanical injury and SGE applications on the test plants. To differentiate the unique role of BPHs SGE, a mechanical injury control group was used. Third, investigating the effect of SGE instead of salivary secretions collected from artificial diets permitted inclusion and assessment of a broader spectrum of salivary compounds. Diet-based saliva collection is greatly limited, due to the fact that diets lack the chemical complexity of plant phloem sap and because compounds secreted into diets degrade rapidly.

In summary, BPH-rice interactions are governed by the interplay of several factors such as microclimate, plant/soil nutrition, plant age, BPHs feeding behaviour and population size. The objective of this study was to investigate one of these factors, that of BPH saliva and its mediated effects on rice responses. Facing a rather challenging and difficult task of studying the role of saliva on plant responses, the current study has approached this through analyses of the effects of SGE application on the rice transcriptome network. Overall, results demonstrated that BPHs SGE modulates rice systemic responses at the mRNA level. Numerous ESTs with functions related to primary metabolism and transport found in the up-regulated pool suggest reprogramming of the host plant transcriptome to enhance host nutrient status. Additional support for this conclusion is provided by the fact that 58% of the up-regulated and 28% from the down-regulated ESTs have similar expression patterns (either up-regulation or down-regulated) to previously studied orthologous SAGs. Furthermore, SGE affects the rice transcriptome network at multiple levels (signaling and defense, transcription, translation and regulation), so as to possibly affect plant signaling/defence responses, neutralize toxic products and promote favourable conditions for hoppers survival. When current results are applied onto ‘whole plant’ scale, it demonstrates that following application, SGE has an almost immediate effect on the distant parts of the host plant so as to precondition these sites in a manner that will sustain and promote BPHs survival. Thus, it is emerging that BPHs saliva has a potential ‘investment’ role, whose future ‘returns’ will enable hoppers survival on the host plant.

## Supporting Information

S1 Fig Comparative analysis of ESTs representation in the two libraries. According to their primary function (A) ESTs from both pools were classified into six functional groups: 1-primary metabolism, 2-transcription/translation/regulation, 3-signaling/defense, 4-transport, 5-other, 6-unknown. Further classification (B) was carried out to identify ESTs with similar expression patterns to orthologous senescence associated genes (SAGs). Based on previous information of SAGs expression patterns during senescence, this study has identified that 58% of the up-regulated and 47% of the down-regulated ESTs are related to orthologous SAGs. However, in the down-regulated pool, only 28% of ESTs have similar expression patterns with down-regulated SAGs. The latter provides further support for the main conclusion in this study, which is that BPHs SGE affects rice transcriptome in such a way as to enhance host nutrient turnover (senescence-like mechanisms) which will have a positive impact on hoppers feeding and survival.
(TIF)

S1 Table Gene name, accession number, primer sequence and amount of total RNA used in each reaction. (DOCX)

S2 Table List of BPH SGE-responsive systemically down-regulated genes in TN1 rice variety. Reference information for genes related to previously studied SAGs is included. SAGs may have variable expression patterns (U, up-regulated; D, down-regulated) during different stages of senescence.
(DOCX)
