# Supplementary figures and images for: Application of Brown Planthopper Salivary Gland Extract to Rice Plants Induces Systemic Host mRNA Patterns Associated with Nutrient Remobilization
Source: PLoS One. 2015 Dec 7;10(12):e0141769. doi: 10.1371/journal.pone.0141769 (PMC4671554; doi:10.1371/journal.pone.0141769)

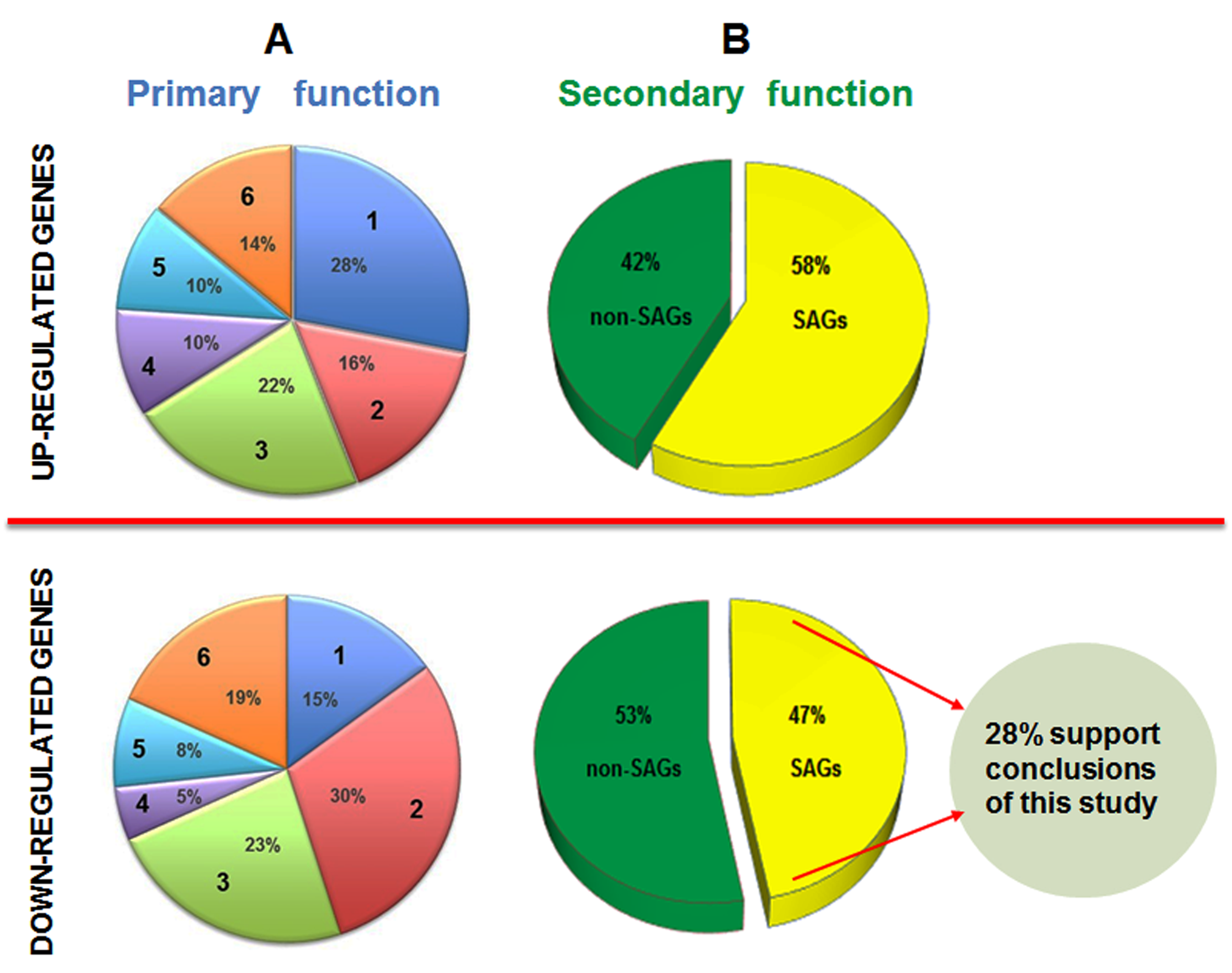

Supplement: S1 Fig — According to their primary function (A) ESTs from both pools were classified into six functional groups: 1-primary metabolism, 2-transcription/translation/regulation, 3-signaling/defense, 4-transport, 5-other, 6-unknown. Further classification (B) was carried out to identify ESTs with similar expression patterns to orthologous senescence associated genes (SAGs). Based on previous information of SAGs expression patterns during senescence, this study has identified that 58% of the up-regulated and 47% of the down-regulated ESTs are related to orthologous SAGs. However, in the down-regulated pool, only 28% of ESTs have similar expression patterns with down-regulated SAGs. The latter provides further support for the main conclusion in this study, which is that BPHs SGE affects rice transcriptome in such a way as to enhance host nutrient turnover (senescence-like mechanisms) which will have a positive impact on hoppers feeding and survival. (TIF) [file pone.0141769.s001.tif]
