# Supplementary material for: Application of Brown Planthopper Salivary Gland Extract to Rice Plants Induces Systemic Host mRNA Patterns Associated with Nutrient Remobilization
Source: PLoS One. 2015 Dec 7;10(12):e0141769. doi: 10.1371/journal.pone.0141769 (PMC4671554; doi:10.1371/journal.pone.0141769)
Supplement: S1 Table — (DOCX) [file pone.0141769.s002.docx]

| Gene name | Accession number | Forward - 5’-3’ | Reverse - 5’-3’ | | RNA ng |
| --- | --- | --- | --- | --- | --- |
| Glutamine synthetase | X14246.1 | GTCATCTTATACCCACAGGCTA | | AGCCTGCTCCGTTATTCTCTCA | 700 |
| Asparagine synthetase | KC140125.1 | AGAGCAAGCACCCCAATGTTCT | | TCAATCCATCAATCCAACTGTA | 700 |
| Amino acid trans | NM_001052691.1 | TCATTGAGATACACATCTGCA | | AGATGGCAGCAAGATAAATTA | 500 |
| Glyoxalase I | GQ848063.1 | TGACTATAAGTATACCATTGCCAT | | TAGCACAGAACTTGGGCCATCTCT | 400 |
| Calcium/proton antiporter | AY156513.1 | ATGAGGATGCAGCAGAGGAAGA | | TACTGTTATGAAGAGCGTTGCTGT | 500 |
| Dehydroascorbate reductase | AB037970.1 | TCTCTTGTCACCCCTCCTGAGTAT | | ATCCGCAATCAGGTGCTCCTTA | 400 |
| 18S | AF069218.1 | AGTGTCTGGTAATTGGAATGAG | | TGTCAATCCTTGCTATGTCTG | 100 |

**S1 Table**
