# Supplementary material for: Application of Brown Planthopper Salivary Gland Extract to Rice Plants Induces Systemic Host mRNA Patterns Associated with Nutrient Remobilization
Source: PLoS One. 2015 Dec 7;10(12):e0141769. doi: 10.1371/journal.pone.0141769 (PMC4671554; doi:10.1371/journal.pone.0141769)
Supplement: S2 Table — Reference information for genes related to previously studied SAGs is included. SAGs may have variable expression patterns (U, up-regulated; D, down-regulated) during different stages of senescence. (DOCX) [file pone.0141769.s003.docx]

| **Group** | **Gene name** | **Gene locus** | **SAG** |
| --- | --- | --- | --- |
|  |  |  | **Ref.** |
|  |  |  |  |
|  | 01. Thylakoid formation 1, chloroplast | LOC_Os07g37250.1 | [D, 32] |
|  | 02. Carbamoyl-phosphate synthase large chain | LOC_Os01g38970.1 | [n/a] |
| **Primary** | 03. 1,4-alpha-glucan-branching enzyme | LOC_Os06g51084.6 | [U-D, 29] |
| **metabolism** | 04. ACT domain-containing protein | LOC_Os12g05650.1 | [n/a] |
|  | 05. Alpha-glucan phosphorylase | LOC_Os01g63270.1 | [n/a] |
|  | 06. Ribulose-phosphate 3-epimerase | LOC_Os03g07300.1 | [U, 29] |
|  | 07. Beta-galactosidase | LOC_Os01g72340.1 | [D-U, 29] |
|  | 08. Phosphoglucomutase | LOC_Os03g50480.2 | [D-U, 29] |
|  | 09. Acyltransferase | LOC_Os11g41900.1 | [D, 29] |
|  |  |  |  |
|  |  |  |  |
|  | 10. tRNA synthetase class I | LOC_Os01g03020.1 | [n/a] |
|  | 11. Transcription initiation factor TFIID, subunit 10 | LOC_Os09g26180.1 | [n/a] |
|  | 12. BTBZ2 | LOC_Os01g68020.1 | [n/a] |
|  | 13. RNA recognition motif containing protein | LOC_Os03g18720.1 | [n/a] |
|  | 14. ATP synthase | LOC_Os01g51380.2 | [D-U, 29] |
| **Transcription/** | 15. Peptidyl-prolyl cis-trans isomerase | LOC_Os02g02890.1 | [D, 29] |
| **Translation/** | 16. T-complex protein 11 | LOC_Os04g35840.1 | [U, 28] |
| **Regulation** | 17. Peptidyl-tRNA hydrolase | LOC_Os01g49900.1 | [D, 29] |
|  | 18. Agenet domain-containing protein | LOC_Os07g41640.3 | [n/a] |
|  | 19. Growth regulating factor protein | LOC_Os07g28430.1 | [n/a] |
|  | 20. B-box zinc finger family protein | LOC_Os06g05890.1 | [U-D, 29] |
|  | 21. RNA polymerase sigma factor | LOC_Os05g51150.3 | [U-D, 29] |
|  | 22. Pre-mRNA-processing factor 39 | LOC_Os03G11200.1 | [n/a] |
|  | 23. Zinc finger CCCH type domain protein | LOC_Os06g21390.1 | [n/a] |
|  | 24. Chaperone protein dnaJ | LOC_Os04g46390.2 | [U, 29] |
|  | 25. Homeobox protein knotted-1 | LOC_Os02g08544.1 | [U, 28] |
|  | 26. Pirin | LOC_Os03g62790.1 | [n/a] |
|  | 27. Signal peptide peptidase | LOC_Os05g36070.2 | [U-D, 28] |
|  |  |  |  |
|  |  |  |  |
|  | 28. Aconitate hydratase protein | LOC_Os08g09200.1 | [D, 29] |
|  | 29. Protein phosphatase 2C | LOC_Os02g27220.1 | [U-D, 29] |
|  | 30. Oxidoreductase | LOC_Os06g30390.1 | [n/a] |
|  | 31. DXR | LOC_Os01g01710.2 | [D, 29] |
| **Signaling/** | 32. Caleosin related protein | LOC_Os02g50174.1 | [U, 29] |
| **Defense** | 33. NADPH-dependent FMN reductase | LOC_Os05g42190.1 | [U, 29] |
|  | 34. CrRLK1L homolog | LOC_Os05g25370.1 | [U-D, 29] |
|  | 35. Pentatricopeptide containing protein | LOC_Os02g07360.1 | [n/a] |
|  | 36. Kinase | LOC_Os02g45130.2 | [U, 28] |
|  | 37. Glutamate--cysteine ligase | LOC_Os05g03820.1 | [n/a] |
|  | 38. Phototropin – kinase activity | LOC_Os11g01140.3 | [D-U, 29] |
|  | 39. Disease resistance protein | LOC_Os01g06790.1 | [U, 28] |
|  | 40. Protein kinase-like (PK-like) | LOC_Os02g14130.1 | [U, 28] |
|  | 41. Amine oxidase | LOC_Os04g57560.1 | [D-U, 29] |
|  |  |  |  |
|  |  |  |  |
|  | 42. Mitochondrial carrier protein | LOC_Os09g33470.1 | [U, 28] |
| **Transport** | 43. GHMP kinases ATP-binding protein | LOC_Os01g58790.1 | [n/a] |
|  | 44. ER-Golgi intermediate compartment protein | LOC_Os03g20520.1 | [n/a] |
|  |  |  |  |
|  |  |  |  |
|  | 45. GTP cyclohydrolase 1 | LOC_Os04g56710.1 | [n/a] |
|  | 46. HEAT repeat family protein | LOC_Os01g60040.2 | [n/a] |
| **Other** | 47. Zinc-binding alcohol dehydrogenase | LOC_Os09g28570.3 | [n/a] |
|  | 48. Reticulon domain containing protein | LOC_Os07g04910.1 | [U, 28] |
|  | 49. C2 domain containing protein | LOC_Os01g03820.1 | [n/a] |
|  |  |  |  |
|  |  |  |  |
|  | 50. Expressed protein | LOC_Os06g38970.1 | [n/a] |
|  | 51. Expressed protein | LOC_Os06g50350.1 | [n/a] |
|  | 52. Expressed protein | LOC_Os03g41229.1 | [n/a] |
|  | 53. Expressed protein | NCBI -NM_001066226 | [n/a] |
| **Unknown** | 54. Expressed protein | LOC_Os01g72990.2 | [n/a] |
|  | 55. Expressed protein | LOC_Os09g01000.1 | [n/a] |
|  | 56. Expressed protein | LOC_Os01g68300.1 | [n/a] |
|  | 57. Expressed protein | LOC_Os01g06270.2 | [n/a] |
|  | 58. Expressed protein | LOC_Os12g37860.1 | [n/a] |
|  | 59.Expressed protein | LOC_Os01g57968.1 | [n/a] |
|  | 60. Expressed protein | LOC_Os02g55670.1 | [n/a] |
